# Isolation and Identification of Inter-Correlated Genes from the Invasive Sun Corals *Tubastraea Coccinea* and *Tubastraea Tagusensis* (Scleractinia, Cnidaria)

**DOI:** 10.3390/ijms26157235

**Published:** 2025-07-26

**Authors:** Maria Costantini, Fulvia Guida, Carolina G. Amorim, Lucas B. da Nóbrega, Roberta Esposito, Valerio Zupo, Beatriz G. Fleury

**Affiliations:** 1 Department of Ecosustainable Marine Biotechnology, Stazione Zoologica Anton Dohrn, Villa Comunale, 80121 Naples, Italy; fuffyguida12@gmail.com (F.G.); roberta.esposito@szn.it (R.E.); 2 Department of Ecosustainable Marine Biotechnology, Stazione Zoologica Anton Dohrn, Ischia Marine Center, 80077 Ischia, Italy; valerio.zupo@szn.it; 3 Department of Biology, University of Naples Federico II, Via Cinthia Monte Sant’Angelo, 80100 Naples, Italy; 4 Graduate Program in Ecology and Evolution, University of the State of Rio de Janeiro, Rua São Francisco Xavier 524, PHLC Sala 224, Rio de Janeiro 20550-013, RJ, Brazil; carolgdeamorim@gmail.com (C.G.A.); nobregalucas@gmail.com (L.B.d.N.); 5 Ecology Department, University of State of the Rio de Janeiro, Rua São Francisco Xavier 524, PHLC Sala 220, Rio de Janeiro 20550-900, RJ, Brazil; bgfleury@gmail.com

**Keywords:** gene, gene network, interactomic analysis, RNA, sun coral

## Abstract

*Tubastraea coccinea* and *T. tagusensis*, commonly known as sun corals, are two species of stony corals (Scleractinia, Dendrophylliidae) native to the Indo-Pacific region (*T. coccinea*) and the Galapagos Islands (*T. tagusensis*), respectively. They are considered highly invasive species, particularly in the Western Atlantic Ocean, due to high adaptability to various ecological conditions and notable resilience. Given their demonstrated invasiveness, it is important to delve into their physiology and the molecular bases supporting their resilience. However, to date, only a few molecular tools are available for the study of these organisms. The primary objective of the present study was the development of an efficient RNA extraction protocol for *Tubastraea coccinea* and *T.a tagusensis* samples collected off Ilha Grande Bay, Rio de Janeiro (Brazil). The quantity of isolated RNA was evaluated using NanoDrop, while its purity and quality were determined by evaluating the A260/A280 and A260/230 ratios. Subsequently, based on genes known for *T. coccinea*, two housekeeping genes and seven stress response-related genes were isolated and characterized, for the first time for both species, using a molecular approach. An interactomic analysis was also conducted, which revealed functional interactions among these genes. This study represents the first report on gene networks in *Tubastraea* spp., opening new perspectives for understanding the chemical ecology and the cellular mechanisms underlying the invasiveness of these species. The results obtained will be useful for ecological conservation purposes, contributing to the formulation of strategies to limit their further expansion.

## 1. Introduction

Several Indo-Pacific scleractinian corals have invaded warm ocean basins in recent decades [1]. Notable examples include the northern Gulf of Mexico [2] and, the southwestern Atlantic Ocean, where they have already established widespread populations. Among these, *Tubastraea micranthus*, *T. coccinea*, and *T. tagusensis* are well documented as introduced and invasive species in the Western Atlantic [3]. Their invasiveness is attributed to their facile settlement on mobile oil and gas platforms, which serve as vectors for their introduction, when relocated [4,5,6]. Introduced as early as 1980 through opportunistic colonization, these species are now widespread along more than 3500 km of coastline [7]. In their final destination, such as Brazilian shallow-waters tropical rocky reefs, an explosive growth phase is frequently observed. This rapid proliferation is facilitated by a lack of natural predators and their strong competitive ability against local corals and other organisms that are not adapted to their invasive presence [8,9,10,11]. Consequently, these invasive corals pose a significant threat to endemic species and negatively impact community structure, functioning [9], and trophic interactions [12,13]. In fact, their densities often reach up to 300 individuals per square meter. While similar geographic invasion patterns are commonly observed, different *Tubastraea* species often exhibit variable depth ranges. Depth limitations may be critical for species requiring light for their trophism, whereas azooxanthellate species, such as *T. coccinea* and *T. tagusensis*, may be primarily limited by food availability. *Tubastraea coccinea* is also abundant on artificial substrata in the Gulf of Mexico [14,15,16,17], frequently occurring in high abundances on floating platforms (up to hundreds of thousands of colonies per platform). It may also be found in lower abundances [18,19,20] on deep banks in the northern Gulf of Mexico. Furthermore, it has been reported on the coral reefs of the Flower Garden Banks [21,22], where it appears to be a cryptic species.

The success of alien species invasions is largely dependent on their reproductive rates, but population dynamic features, such as specific growth rates and mortality, are other key elements to forecast their fitness in a new area. Both *T. tagusensis* and *T. coccinea* exhibit budding, simple colony growth, and asexual planulae production [23,24]. The also commonly undergo sexual reproduction, emitting gametes all year-round, which is crucial for population dispersal far from the original settlement site. Indeed, their larval dispersal capabilities are remarkable [25]. Interestingly, even the smallest colonies (comprising a few polyps) of *T. coccinea* may produce viable gametes. Their planulae develop in only six weeks, subsequently settling and metamorphosing within three days. While planulae are primarily released from March through July, varying by site, both species are considered to be highly fecund [26]. Notably, the Brazilian coast colonies of these alien corals have been observed to produce oocytes throughout the year, with two distinct reproductive peaks, each lasting 3–4 months. The reproductive biology of these species contributes to their successful colonization, diffusion, and expansion into new habitats, leading to their population consolidation [27].

In addition, the physical characteristics of the new environment, coupled with their physiological flexibility and trophic adaptability, are crucial factors in determining the ability of sessile organisms to successfully compete with native species for space and trophic resources. For instance, the Indo-Pacific species *T. coccinea* [28] was first introduced in the waters off Puerto Rico in 1943. It subsequently spread to Curaçao in 1948 [21,29], located in the Netherlands Antilles. Its expansion continued in the late 1990s up to the year 2000, reaching Belize and Mexico [30], Venezuela, the northern Gulf of Mexico, and the Florida Keys [1,14,21,24]. Among the most recently colonized sites are Brazil [3], Colombia, Panama, the Bahamas, and the Lesser and Greater Antilles [30,31]. Once the population of an introduced species becomes established in a specific location, it can expand quickly and widely, becoming invasive and rendering eradication attempts largely difficult [32].

Sessile epibenthic organisms employ specific mechanisms to compete for space, including chemical inhibition of competitors. Indeed, allelopathy has been demonstrated in several terrestrial plants [33,34], as well as in Indo-Pacific alcyonacean soft corals, on the Great Barrier Reef [35,36,37,38,39]. Competition between corals and algae has also been observed [40]. Understanding the genic resources facilitating these relationships may be key to reduce the expansion of invasive species. Thus, to determine the factors promoting the adaptability and spread of *Tubastraea* spp., and the genic resources sustaining the adaptability of these impressive competitors for space [9,10,11,41,42], it is essential to establish efficient methods for RNA extraction, and to analyze the main physiologic pathways. Molecular biology approaches are increasingly helping to define specific ecological questions related to biogeography, genomics, conservation genetics, and behavioral ecology. Here, an RNA extraction method from *T. coccinea* and *T. tagusensis* colonies was developed to obtain high yields both in quality and quantity. For the first time, two housekeeping genes and seven stress response-related genes were isolated and identified in these two corals using a molecular approach. We also conducted an interactomic analysis on these genes to ascertain their inter-correlation and to describe the specific gene networks in which they are involved.

## 2. Results and Discussion

Extractions yielded sufficient amounts of total RNA from both *T. coccinea* and *T. tagusensis* tissues (Figure S1). The quantity, purity (A260/280 and A260/230 ratios), and integrity (RIN values) of the extracted RNA met the requirements for Next Generation Sequencing approaches, such as transcriptome and genome sequencing, as detailed in Table 1.

**Figure 1:**
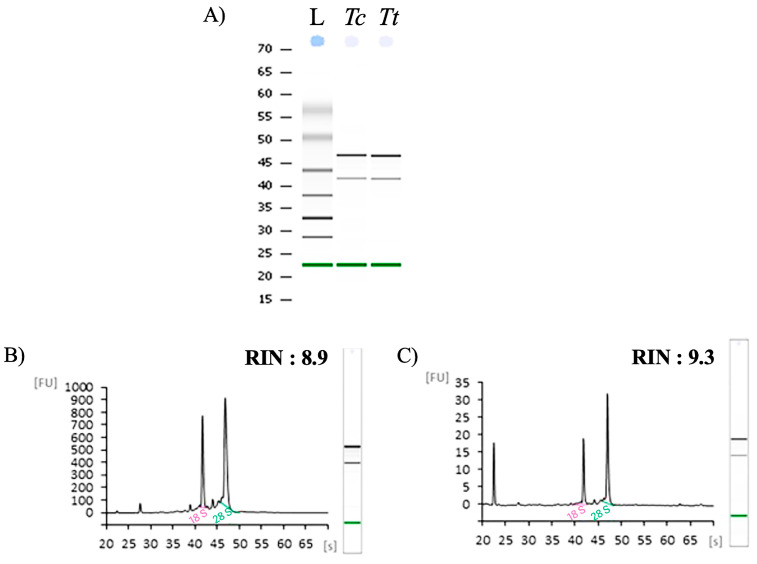
Bioanalyzer Agilent electrophoresis runs for the (**A**)) RNA extracted from *T. coccinea* (*Tc*) and *T. tagusensis* (*Tt*) tissue. In the first lane of the run the ladder (L) is reported. Agilent Bioanlyzer electropherograms of RNA extracted from (**B**)) *Tc* and (**C**)) *Tt* Relative Fluorescent Unit (FU) and seconds of migration (s) of RNA samples. RIN values are also reported for the two RNA analyzed.

Bioanalyzer Agilent electrophoresis runs confirmed the high quality of total RNA extracted from both *T. coccinea* and *T. tagusensis* (Figure 1). Following extraction, cDNA was successfully generated through reverse transcription.

**Figure 2:**
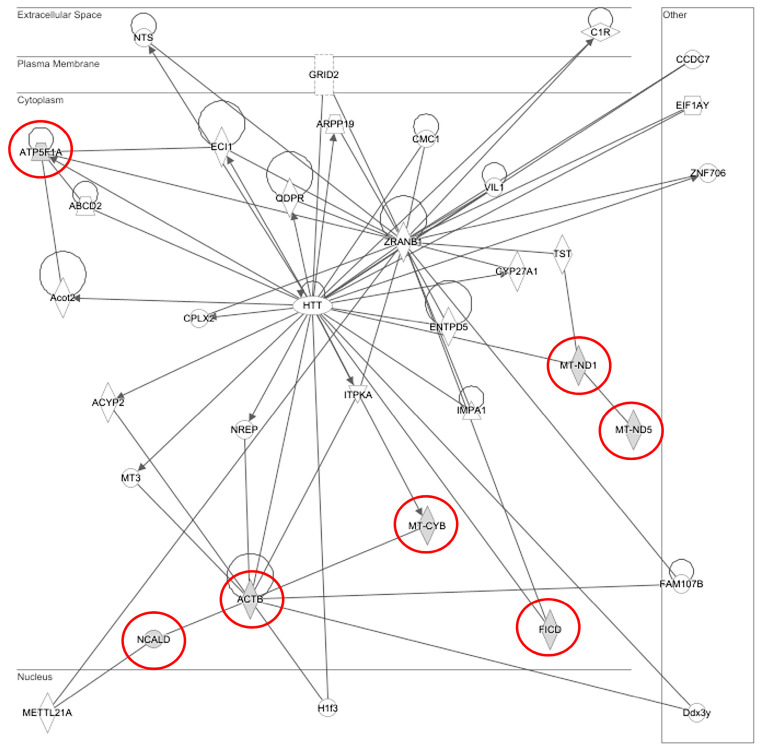
Network of genes involved in apoptosis generated by interactomic analysis performed through *Ingenuity Pathway Analysis* (IPA) software (version 7.1). The genes that were analyzed are highlighted with red circles. The arrows (indicating how a molecule can modulate the expression of the others showed the biological relationships between the genes analyzed. The edges indicated the connections concerning direct relationships.

Here, for the first time, we isolated and identified seven stress-response genes in *T. tagusensis*, based on available sequences for *T. coccinea*, as detailed below. Gene sequences were confirmed through sequencing (Figure S3). An interactomic analysis was performed using *Ingenuity Pathway Analysis* (IPA) on the identified genes from *T. coccinea* to identify functional networks. While this molecular tool primarily supports vertebrate models for analysis, leading to potential differences in coral-specific interactions, the generated network (Figure 2) illustrates functional associations derived from literature data. Consequently, gene interactions likely hold true in corals, but some differences could be still detected. The gene network (Figure 2) connects the seven identified stress-response genes: *Adenosine-monophosphate-protein-transferase* (*AMPt*, in human *FICD*), *Adenosine triphosphate synthase* (*ATPs*, in human *ATP5F1A*), *Beta-actin* (*Beta-act,* in human *ACTB*), *Cytochrome b* (*Cytb,* in human *MT-CYB*), *NADH dehydrogenase subunit 5* (*NADH5,* in human *MT-ND5*), *NADH-ubiquinone oxidoreductase* (*NADHox,* in human *MT-ND1*), and *Neurocalcin-like protein* (*NC,* in human *NCALD*).

Based on their known functions, all these genes are indeed likely involved in stress responses. The DAVID gene functional classification selected KEGG functional pathways that showed statistically significant gene enrichment (*p* < 0.05) for the genes under analysis (Table S1). Our analysis showed that those genes were mainly involved in four KEGG pathways: thermogenesis, electron transport, coupled proton transport, oxidative phosphorylation, and mitochondrion inner membrane. In particular:

*FICD* (human ortholog of AMPt) encodes an enzyme facilitating the transfer of an adenosine monophosphate (AMP) group from ATP to a protein, typically modifying its structure or function [43]. This transfer, occurring on the endoplasmic reticulum (ER) membrane, is involved in various cellular processes including protein activity regulation and signaling. It also regulates IRE1-mediated unfolded protein response and general ER stress response.

*ATP5F1A* (human ortholog of ATPs) encodes the alpha subunit of mitochondrial ATP synthase, a protein complex essential for ATP synthesis within mitochondria [44]. This enzyme is crucial for energy production, making it a key player in cellular metabolism and involved in oxidative stress response.

*ACTB* (human ortholog of Beta-act) is a highly conserved gene responsible for producing actin filaments that form cross-linked networks in the cell cytoplasm [45]. Six different actin proteins exist, involved in cell motility, structure, and integrity. Changes in ACTB expression or function can impact various cellular processes, including those related to stress [46].

*MT-CYB* (human ortholog of Cytb), located in the mitochondrion, contributes to cytochrome-c oxidase activity and is involved in aerobic respiration, positive regulation of vasoconstriction, and the respiratory electron transport chain. It also plays a role in stress response, particularly oxidative stress [47,48].

*MT-ND5* (human ortholog of NADH5) provides instructions for creating NADH dehydrogenase subunit 5, a core subunit of the respiratory chain NADH dehydrogenase (Complex I) in mitochondria. This protein is essential for the electron transport process, which helps generate ATP (energy) within mitochondria [49]. It plays a crucial role in stress response, especially when mutations occur, which can lead to oxidative stress, DNA damage, and neurodegenerative conditions [50].

*MT-ND1* (human ortholog of NADHox) is a mitochondrial gene that codes for NADH dehydrogenase subunit 1, a key component of Complex I located in the inner mitochondrial membrane and involved in the electron transport chain. This protein is essential for oxidative phosphorylation, the process by which mitochondria produce energy [51], and is involved in cellular stress responses, particularly those related to oxidative stress [52].

*NCALD* (human ortholog of NC) encodes a neuronal calcium sensor (NCS) belonging to the family of calcium-binding proteins [53]. This cytosolic protein interacts with clathrin and actin.

### Gene Network Analysis and Hub Genes

Detailed analysis of the network indicated that *ATP5F1A* and *ACTB* genes can be considered HUB genes, suggesting their key role in significant functional nodes within the gene network due to their highest number of relationships with other nodes. Firstly, the seven genes analyzed are functionally interconnected. Specifically, *MT-ND5* interacts with *MT-ND1*. The latter, in turn, interacts with *MT-CYB* via the Huntingtin (HTT) gene (a disease gene linked to Huntington’s disease, a neurodegenerative disorder characterized by loss of striatal neurons) and Inositol-Trisphosphate 3-Kinase A (*IPTKA*; a gene that regulates inositol phosphate metabolism by phosphorylation of the second messenger inositol 1,4,5-trisphosphate, responsible for regulating inositol polyphosphates in signaling [54]). *MT-CYB* is directly linked to *ACTB*, which in turn is functionally related to *NCALD*. *MT-ND1* also interacts with *FICD* via *HTT*, which in turn interacts with Inositol Monophosphatase 1 (*IMPA1*; a gene encoding inositol monophosphatase-1, an enzyme critical for recovery of the inositol cycle, important for both inositol synthesis and the use of inositol polyphosphates generated after receptor activation [55]).

Considering the HUB node *ATP5F1A*, it interacts with the following: i. *Enoyl-CoA Delta Isomerase 1* (*ECI1*), a gene involved in beta-oxidation of unsaturated fatty acids [56]; ii. *ATP Binding Cassette Subfamily D Member 2* (*ABCD2*) encodes a peroxisomal ATP-binding cassette (ABC) transporter, specifically the ATP-binding cassette subfamily D member 2; it is involved in transporting long- and very long-chain fatty acids into peroxisomes [57]; iii. *Acyl-CoA Thioesterase 2* (*Acot2*), which plays a role in fatty acid metabolism, particularly in mitochondria, catalyzing the hydrolysis of acyl-CoAs into free fatty acids and coenzyme A (CoASH), regulating their respective intracellular levels [58].

The second HUB gene *ACTB* has significant interactions with the following: i. *Methyltransferase 21A, HSPA Lysine 2* (*METTL21A*). The gene encodes for protein–lysine methyltransferase that is responsible for the trimethylation of heat shock protein 70 (HSP70), which in turn is involved in cellular stress responses and protein folding [59]. ii. *Family with Sequence Similarity 107 Member B* (*FAM107B*), a gene that functions as a tumor suppressor, as its expression is often diminished in various cancers, leading to tumor development and proliferation [60]. iii. *Neuronal Regeneration Related Protein* (*NREP*), located in cytoplasm and involved in regulation of axon and neuron differentiation [61,62]. iv. *Metallothionein 3* (*MT3*), a gene that encodes the protein metallothionein-3, also known as growth inhibitory factor (GIF), which is a small, cysteine-rich protein, involved in regulating cell growth and the response to oxidative stress [63]. v. *Acylphosphatase 2* (*ACYP2*) encodes an enzyme involved in the regulation of calcium homeostasis, which hydrolyzes the membrane pumps Ca2+/Mg2+-ATPase in sarcoplasmic reticulum of skeletal muscle [64,65].

**Figure 3:**
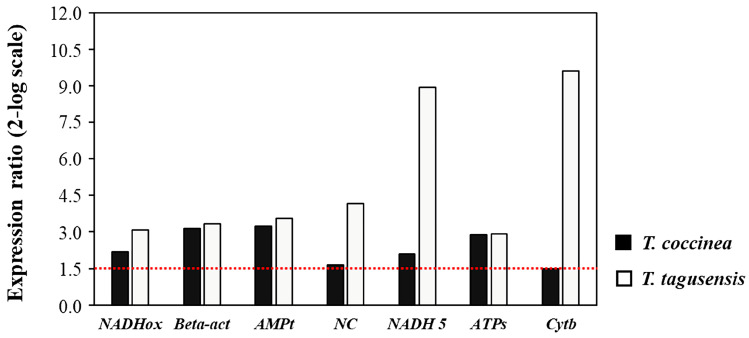
Histograms reported the variation of gene expression (as fold-changes) in *T. coccinea* and *T. tagusensis*. Fold differences greater than ± 1.5 (see dotted horizontal guidelines at values of +1.5 and −1.5) were considered significant.

All these genes were targeted by stress conditions in *T. coccinea* and *T. tagusensis*. Indeed, when analyzed by real-time qPCR comparing laboratory-reared samples with field-collected samples, these seven genes were found to be up-regulated, showing an increase in their expression levels (Figure 3).

Pacific *Tubastraea* species have rapidly expanded throughout the Atlantic Ocean, outcompeting native endemic species and dominating over 95% of the substrate in certain areas. Given the limited genic data available for azooxanthellate corals, Capel et al. [66] made a significant contribution by determining the complete mitochondrial DNA sequences of Atlantic individuals of *T. coccinea* and *T. tagusensis*, which is crucial for understanding their phylogenetic relationships and evolutionary history.

Notably, the genes isolated in this study represent a valuable molecular tool for investigating infochemicals involved in *Tubastraea* species expansion, including chemotactic activities and gene activation. As sun corals achieve invasiveness through high adaptability to various ecological conditions and strong resilience, these biomarkers can facilitate understanding of the chemical ecology of *T. coccinea* and *T. tagusensis.* Furthermore, this knowledge can inform the development of strategies for managing their invasions, potentially by utilizing metabolites produced by other organisms to modulate their ecophysiology and expansion dynamics.

## 3. Materials and Methods

### 3.1. Sample Collection, Preservation, and RNA Extraction

A collection of invasive scleractinian corals, *Tubastraea coccinea* Lesson, 1829 and *T. tagusensis* Wells, 1982 (Cnidaria, Anthozoa), took place at Abraãozinho, Ilha Grande (23°06′53.2″ S, 44°09′57.4” W), Angra dos Reis, Rio de Janeiro, Brazil. *T. coccinea* typically forms nearly spherical colonies, featuring a white corallum and a coenosarc in shades of red to orange. Its skeleton can reach diameters of up to 105 mm, with corallites moderately spaced and extending approximately 3.2 mm above the *coenosteum*. In contrast, *T. tagusensis* develops colonies that are also roughly spherical, often globular and convex in shape, with a yellow coenosarc. Its white coral can grow as large as 150 mm in diameter, and the prominent corallites project approximately 18.5 mm above the *coenosteum* [3,67]. For field samples, single polyps were collected, dissected, and transferred into Eppendorf vessels containing 200 µL of RNAlater. These samples were subsequently frozen at −20 °C until use. For laboratory-acclimated samples, colonies of *T. coccinea* and *T. tagusensis* were held in aquaria with a biological filter and oxygenation at a controlled temperature of 18 °C for approximately 1 h of acclimation. Following this, corals were kept in beakers containing 500 mL of seawater for three hours. Single polyps were then collected, dissected, and transferred into Eppendorf vessels containing 200 µL of RNAlater, as previously described for the field samples.

Total RNA was extracted from three samples using 30 mg of both *T. coccinea* and *T. tagusensis* tissues by RNeasy Mini Kit, following the manufacturer protocol (Qiagen, Austin, TX, USA). RLT/2-ME buffer (10 µL β-mercaptoethanol for each mL of RLT buffer) was added. Samples were homogenized with Tissue Lyser (Qiagen, Austin, TX, USA), using 3 mm sterile aluminum beads at 20.1 Hz for 3 min. RNA extracted was eluted with 30 µL RNase-free water, then stored at −80 °C.

The quantity of total RNA extracted was calculated based on the absorbance at 260 nm, and purity was assessed by the 260/280 and 260/230 nm ratios (NanoDrop spectrophotometer ND-1000 UV–Vis Spectrophotometer; NanoDrop Technologies, Wilmington, DE, USA). The integrity of RNA samples was initially detected by running approximately 300 ng of RNA on a 0.8% agarose gel (Figure S1). RNA integrity was further assessed by running 100–200 ng of RNA samples on a 6000 Nano LabChip in an Agilent Bioanalyzer 2100 (Agilent Technologies, Santa Clara, CA, USA). The RNA integrity number (RIN) value was measured based on the comparison of the areas of 18S rRNA and 28S rRNA, with RIN values greater than 8 indicating non-degraded RNA.

For each sample, 600 ng of total RNA extracted was retrotranscribed with an *iScript cDNA synthesis kit* (Bio-Rad, Milan, Italy), according to the manufacturer’s instructions.

### 3.2. Isolation and Identification of Genes

The sequences of two housekeeping genes and seven genes related to stress response were retrieved from *T. coccinea* using the Taxonomy Browser database (available at https://www.ncbi.nlm.nih.gov/Taxonomy/Browser/wwwtax.cgi (accessed 31 January 2025).). Specific primers were designed on the basis of nucleotide sequences of these genes (Table 2).

For each gene, specific primers were designed on the basis of *T. coccinea* nucleotide sequences and used to amplify the selected fragments also from *T. tagusensis*.

To design primers, factors like length, melting temperature (Tm), guanine/cytosine (GC) content, and avoidance of secondary structures were considered. Primers typically have the following characteristics: i. they are 18–25 base pairs long; ii. they should have a melting temperature between 55–75 °C; iii. they should have GC content of 40–60%; iv. they must minimize self-complementarity and the formation of primer dimers.

Amplification reactions by polymerase chain reaction (PCR) were performed in a 30 µL final volume using *Xtra Taq Pol* (GeneSpin Srl, Milan, Italy). Each reaction contained 6 µL of 10× PCR reaction buffer, 6 µL of 10× 2 mM dNTP, 0.5 µL of 5 U/µLTaq, and 25 pmol/µL of each of primer, template cDNA, and nuclease-free water (Figure S2A,B). The PCR program included a cDNA denaturation step at 95 °C for 5 min, followed by 45 cycles of denaturation at 95 °C for 45 s, 54–60 °C for 1 min, and 72 °C for 1 min, and then a final extension step at 72 °C for 10 min. Amplified fragments were purified from agarose gel using the *QIAquickGel Extraction kit* (Qiagen, Milan, Italy), and their specificity was verified through DNA sequencing. Sequence alignments were performed using *MultAlin* (available at http://multalin.toulouse.inra.fr/multalin/ (accessed on 1 February 2025); Figure S3). The same primers were also successfully used to amplify all target genes from cDNA synthetized from *T. tagusensis*.

Melting curve analysis was performed for each pair of primers to determine their specificity in the amplification reactions. The efficiency (E) values of each primer pair were calculated using the following formula:

E = 10^−1^/slope (1)

where “slope” corresponds to the slope of the standard curve, plotted with the y axis as cycle threshold (Ct) and the x axis as log(quantity).

Standard curves were generated from five serial dilutions (1:1, 1:5, 1:10, 1:50, 1:100) for which cycle threshold (Ct) values were detected and plotted against the logarithm of the corresponding dilution factor (Figure S4). Values close to 2 for the slopes of these curves indicated successful amplification of the PCR fragments. Quantitative PCR (qPCR) reactions were performed in triplicates, each with a final volume of 10 µL. Each reaction contained a final concentration of 0.3 mM for each primer and 2× Optimum qPCR Master Mix with SYBR^®^ Green (GeneSpin). The thermal profile used was as follows: (i) initial cDNA denaturation: 95 °C for 10 min; (ii) amplification (40 cycles): 95 °C for 15 s, followed by 60 °C for 1 min; (iii) final elongation: 72 °C for 5 min; (iv) melting curve analysis: 60 °C to 95 °C, to verify the presence of a single product. Fluorescence was determined using QuantStudio 1.5.2.

### 3.3. Real-Time qPCR Experiments

Molecular investigations were performed on three samples from three individuals each of *T. coccinea* and *T. tagusensis*. The expression level of the seven genes isolated in this work was quantified through real-time quantitative PCR (qPCR), using 2× Quantitative Master Mix with SYBR Green Low Rox (Genespin Srl, Milan, Italy). The following thermal profile was adopted: (i) initial denaturation: 95 °C for 20 s; (ii) amplification (40 cycles): 95 °C for 1 s, followed by 60 °C for 20 s; (iii) final elongation: 95 °C for 15 s, followed by 60 °C for 15 s.

Fluorescence was measured using the Bio-Rad CFX Maestro software 2.3 (Bio-Rad Laboratories, Inc.; Hercules, CA, USA). The expression level of each gene was normalized using the Relative Expression Software Tool (REST; REST-MCS©—version 2, Weihenstephan, Germany), with 18S RNA and 28S RNA as housekeeping genes. Values for the expression levels of the analyzed genes were reported relative to the control. Differences larger than a 1.5-fold change were considered significant (Table S2 in Supplementary Materials for detailed values).

### 3.4. Interactomic Analysis

Network analysis was performed using Ingenuity Pathway Analysis Version 7.1 (IPA, Ingenuity Systems, Inc., Redwood City, CA, USA). The analysis employed the Causal Network Analysis algorithm and the Ingenuity Knowledge Base database, with an adjusted *p*-value of *p* < 0.05. This tool facilitated the identification of relationships based on associated functions and data mining from various sources, including experimental studies reported in peer-reviewed articles and PubMed abstracts, public databases (e.g., NCBI, GO, OMIM, UniProt, KEGG, Reactome), and experimentally verified and manually curated by experts. The list of genes was transformed into a set of relevant networks, based on the Ingenuity Pathways Knowledge Base (IPKB). Networks were graphically displayed with nodes representing the genes of interest, and the edges corresponding to the biological relationships between nodes [68]. Nodes with a large number of connections were identified as HUB nodes. Since *Tubastraea* genes are not directly annotated in the IPA database, the names of human orthologous genes were used to search for the genes of interest (Table 3).

Functional annotation analysis was also performed using the Database for Annotation, Visualization and Integrated Discovery (DAVID; https://david.ncifcrf.gov/ (accessed on 5 July 2025)). This widely used online bioinformatics platform provides a comprehensive set of functional annotation tools for analyzing large lists of genes or proteins, thereby aiding in the understanding of their biological significance. The analysis specifically utilized the Kyoto Encyclopedia of Genes and Genomes (KEGG) database, a comprehensive bioinformatics resource that integrates molecular-level information (derived from genome sequencing and other high-throughput experimental technologies) to elucidate high-level functions and utilities of biological systems.

## Figures and Tables

**Table 1:** Total RNA quantity (μg), purity (A260/280 and A260/230), and integrity (RIN values) from *T. coccinea* and *T. tagusensis* tissues.

Sample	RNA Quantity (µg)	A_260_/A_230_	A_260_/A_280_	RIN
RNA_*Tc*	14.8	2.25	1.98	9.3
RNA_*Tt*	7.5	2.15	1.97	10.0

**Table 2:** Seven genes involved in stress response isolated from *T. coccinea* using Taxonomy Browser database (with acronym, gene name, primer names and sequences, and lengths of amplified fragments). Two housekeeping genes are also reported.

Gene Type	Acronym	Acc. Number	Gene Name	Primer	Sequence 5′>3′	Fragment Lenght (bp)
**Housekeeping**	*18S RNA*	LT630999	*18S ribosomal RNA*	18S_Tc_F1	CATAGTAACTGATCGAATCGC	185
				18S_Tc_R1	CGCGCCTGCTGCCTTCCTTG	
	*28S RNA*	AF265625	*28S ribosomal RNA*	28S_Tc_F1	GCGGAGGAAAAGAAACTAAC	195
				28S_Tc_R1	GTCGGCCGTGCCACAAACGG	
**Stress**	*NADHox*	MW139629	*NADH-ubiquinone oxidoreductase*	NADHox_Tc_F1	GGGTTGGTTTATGTTCTTATC	200
				NADHox_Tc_R1	GCTAGATGGGGCAGAAACAAC	
	*Beta-act*	MW139511	*Beta-actin*	Beta-act_Tc_F1	CACCAGCATTTTATGTCGCC	178
				Beta-act_Tc_R1	CTTCATGAGGTAGTCGGTC	
	*AMPt*	MW139419	*Adenosine-monophosphate-protein-transferase*	AMPt_Tc_F1	CACTGTGAGTGATGTTCTTG	170
				AMPt_Tc_R1	CTCTGGATAACAGCCAGTC	
	*NC*	MW110554	*Neurocalcin-like protein gene*	NC_Tc_F1	CAGAGCTCAAAGAATGGTAC	175
				NC_Tc_R1	GAAATCAATAGTGCCATCGTC	
	*NADH5*	OQ697663	*NADH dehydrogenase subunit 5*	NADH5_Tc_F1	CTCATATTCCTCGCTTTATGTC	188
				NADH5_Tc_R1	GACTAACATGGCTTTTATGGC	
	*ATPs*	OQ697278	*Adenosine triphosphate synthase*	ATPs_Tc_F1	GTGGCTCTGATCGCCTTGAC	204
				ATPs_Tc_R1	GAAGAGAGAGACAATAAAAGG	
	*Cytb*	OQ696950	*Cytochrome b*	Cb_Tc_F1	GCCACTGCGCAAAGAGAATC	167
				Cb_Tc_R1	CTGCACAATAATGCATGGAC	

**Table 3:** The corresponding names of *T. coccinea* and human genes are reported.

Gene Name	*T. coccinea*	Human
*adenosine-monophosphate-protein-transferase*	*AMPt*	*FICD*
*ATP synthase*	*ATPs*	*ATP5F1A*
*beta-actin*	*Beta-act*	*ACTB*
*cytochrome b*	*Cytb*	*MT-CYB*
*NADH dehydrogenase subunit 5*	*NADH5*	*MT-ND5*
*NADH-ubiquinone oxidoreductase*	*NADHox*	*ND1*
*neurocalcin-like protein*	*NC*	*NCALD*

## Data Availability

The original contributions presented in this study are included in the article/Supplementary Material. Further inquiries can be directed to the corresponding authors.

## Supplementary Materials

The following supporting information can be downloaded at: https://www.mdpi.com/article/10.3390/ijms26157235/s1.
