# Supplementary material for: Isolation and Identification of Inter-Correlated Genes from the Invasive Sun Corals Tubastraea Coccinea and Tubastraea Tagusensis (Scleractinia, Cnidaria)
Source: Int J Mol Sci. 2025 Jul 26;26(15):7235. doi: 10.3390/ijms26157235 (PMC12347335; doi:10.3390/ijms26157235)
Supplement: Supplementary file 1 [file ijms-26-07235-s001.zip › ijms-3703057-supplementary.pdf]

Figure S1. 0.8% agarose gel on which RNA extracted from 30 mg of *T. coccinea* (*Tc*) and *T. tagusensis* (*Tt*) tissues using RNeasy Mini Kit. About 300 ng were loaded on the agarose gel.

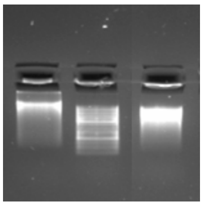

Wells order:

1) RNA *Tc*

2) Ladder plus 100 bp

3) RNA *Tg*

Figure S2. 1.5% agarose gel on which the PCR-amplified fragments for the genes of A) *T. coccinea* (*Tc*) and *T. tagusensis* (*Tt*) were analyzed. The information on the primer pairs used for PCR is reported in **Table 1**. The primer pairs were tested at two annealing temperatures (54°C and 58°C) in order to detect their specificity. Negative controls correspond to PCR in which the cDNA was not adde in the PCR reaction.

A) *T. coccinea*

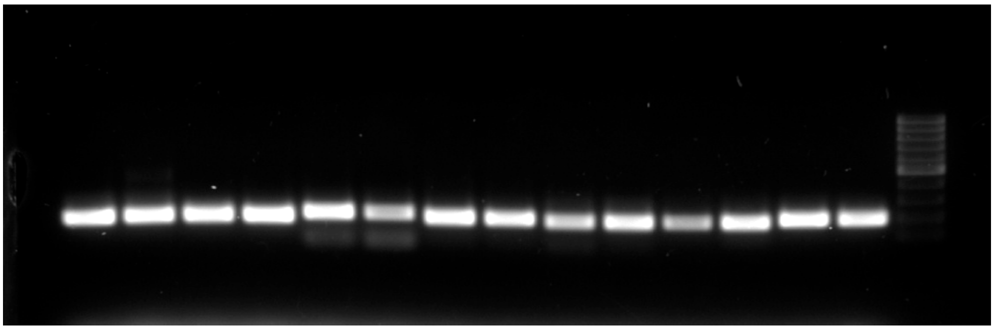

Wells order:

1) *Tc_18S*_F1-R1 54°C
2) *Tc _18S*_F1-R1 58°C
3) *Tc _28S*_F1-R1 54°C
4) *Tc _28S*_F1-R1 58°C
5) *Tc _NADH ox*_F1-R1 54°C
6) *Tc _NADH ox*_F1-R1 58°C
7) *Tc _Beta-act*_F1-R1 54°C
8) *Tc _Beta-act*_F1-R1 58°C
9) *Tc_AMPt*_F1-R1 54°C
10) *Tc_AMPt*_F1-R1 58°C
11) *Tc_ NC*_ F1-R1 54°C
12) *Tc_ NC*_ F1-R1 58°C
13) *Tc_ NADH5*_ F1-R1 54°C
14) *Tc_NADH5*_F1-R1 58°C
15) Ladder plus 100 bp

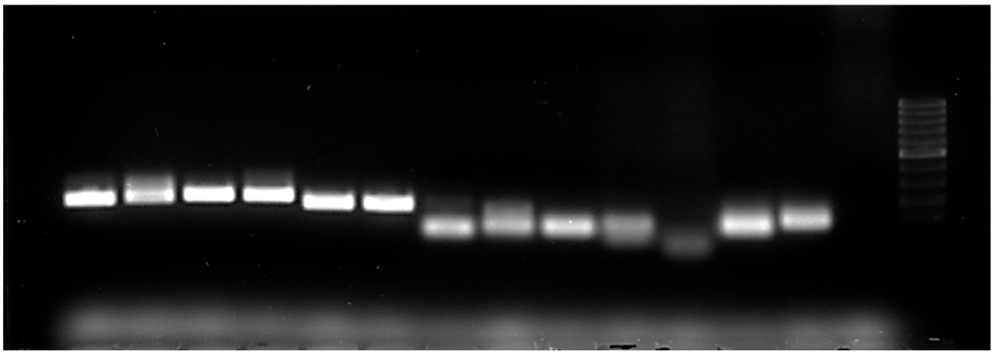

Wells order:

1) *Tc_Cytc*_F1-R1 54°C

9) Negative control *Tc* _NADH ox_F1-R1

2) *Tc _Cytc*_F1-R1 58°C

3) *Tc _ATPs*_F1-R1 54°C

4) *Tc _ATPs*_F1-R1 58°C

5) *Tc _Cytb*_F1-R1 54°C

6) *Tc _Cytb*_F1-R1 58°C

7) Negative control *Tc _18S*_F1-R1

8) Negative control *Tc _28S_F1-R1*

10) Negative control *Tc* _Beta-act_F1-R1

11) Negative control *Tc _AMPt_F1-R1*

12) Negative control *Tc _NC_F1-R1*

13) Negative control *Tc _NADH5_F1-R1*

14) Negative control *Tc _Cytc_F1-R1*

15) Ladder plus 100 bp

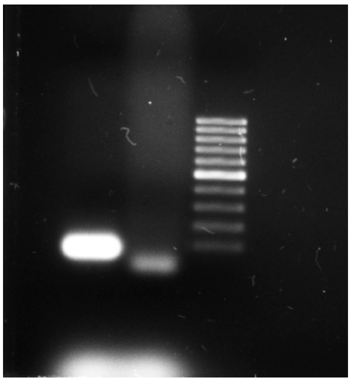

Wells order:

1) Negative control *Tc _ATPs*_F1-R1

2) Negative control *Tc _Cytb*_F1-R1

3) Ladder plus 100 bp

B) *T. tagusensis*

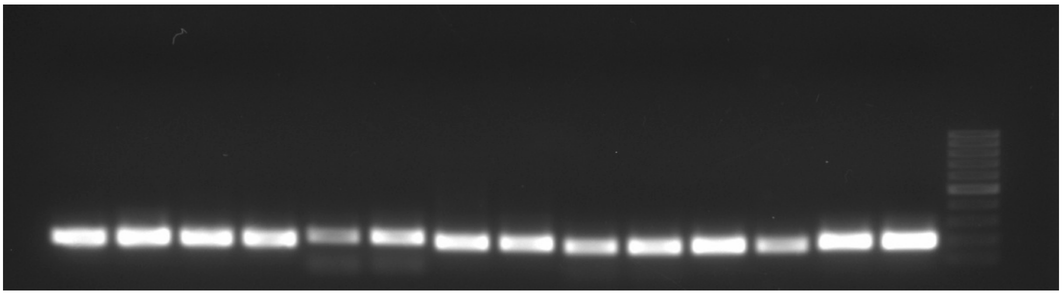

Wells order:

1) *Tt*_18S_F1-R1 54°C

2) *Tt _18S*_F1-R1 58°C

9) *Tt*_AMPt_F1-R1 54°C

10) *Tt_AMPt*_F1-R1 58°C

3) *Tt _28S_*F1-R1 54°C

4) *Tt _28S_*F1-R1 58°C

5) *Tt _NADH ox_*F1-R1 54°C

6) *Tt _NADH ox_*F1-R1 58°C

7) *Tt _Beta-act_*F1-R1 54°C

8) *Tt _Beta-act_*F1-R1 58°C

11) *Tt_ NC_* F1-R1 54°C

12) *Tt_ NC_* F1-R1 58°C

13) *Tt_ NADH5_* F1-R1 54°C

14) *Tt_NADH5_*F1-R1 58°C

15) Ladder plus 100 bp

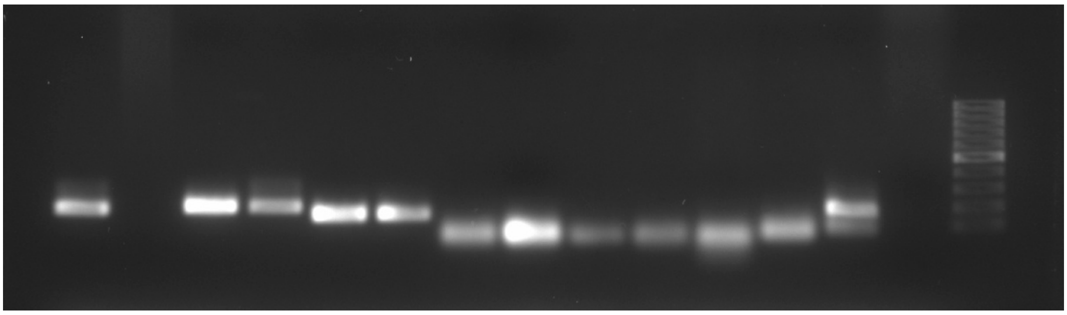

Wells order:

1) *Tt_Cytc_*F1-R1 54°C

2) *Tt _Cytc_*F1-R1 58°C

3) *Tt _ATPs_*F1-R1 54°C

4) *Tt _ATPs_*F1-R1 58°C

5) *Tt _Cytb_*F1-R1 54°C

6) *Tt _Cytb_*F1-R1 58°C

9) Negative control *Tt _NADH ox_*F1-R1

10) Negative control *Tt _Beta-act_*F1-R1

11) Negative control *Tt _AMPt_*F1-R1

12) Negative control *Tt _NC_*F1-R1

13) Negative control *Tt _NADH5_*F1-R1

14) Negative control *Tt*

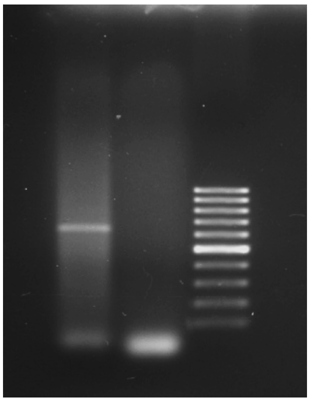

Wells order:

1) Negative control *Tt _ATPs_*F1-R1

2) Negative control *Tt* _*Cytb*_F1-R1

3) *Ladder plus 100 bp*

Figure S3. Alignment obtained with Multalin program, between the complete sequences of the coral *T. coccinea* genes and the fragments amplified by PCR in this work, as well as the primers forward and reverse.

18S RNA (F1-R1)

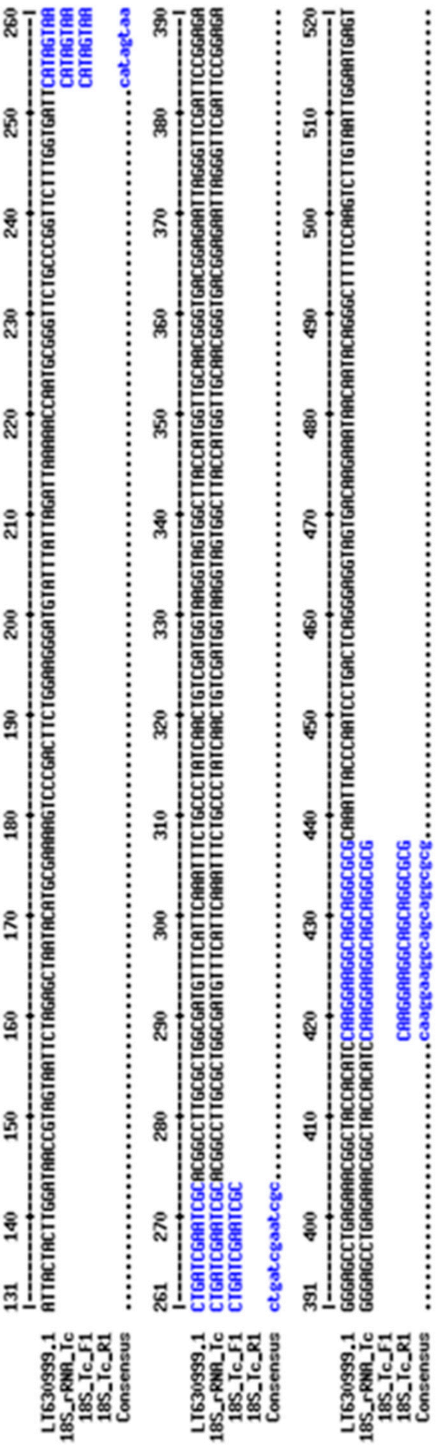

Alignment between the complete sequence of 18S rRNA gene (LT630999.1), the fragment of 18S bases amplified by *T. coccinea*'s cDNA, the forward oligonucleotide 18S_Tc_F1 and the reverse oligonucleotide 18S_Tc_R1, done by using the program MultAlin.

28S RNA (F1-R1)

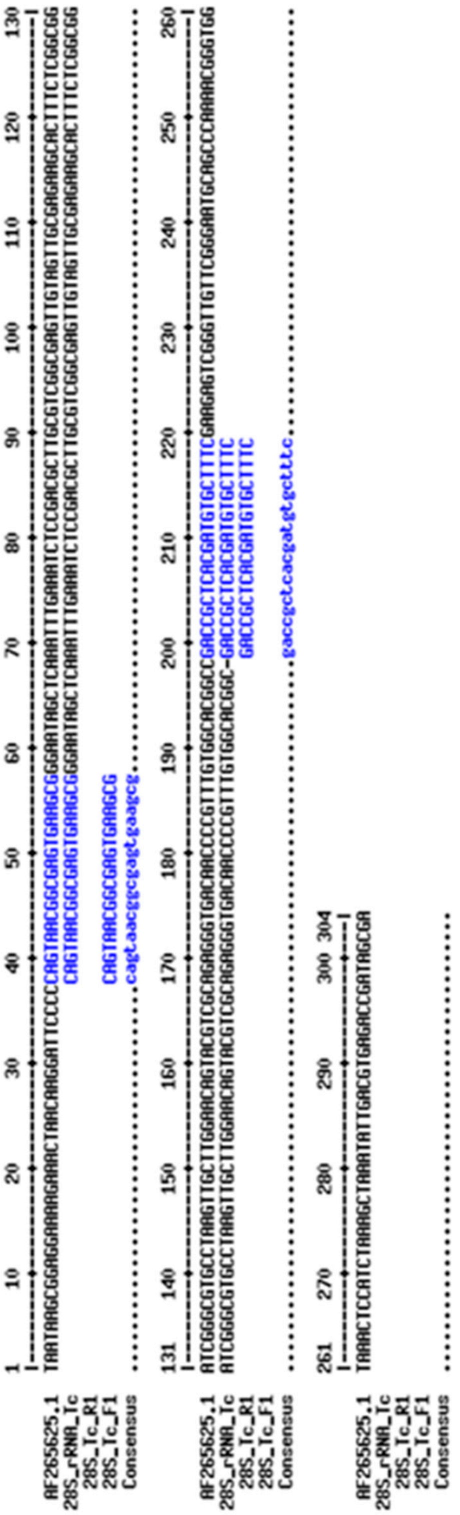

Alignment between the complete sequence of 28S rRNA gene (AF265625.1), the fragment of 195 bases amplified by *T. coccinea*'s cDNA, the forward oligonucleotide 28S_Tc_F1 and the reverse oligonucleotide 28S_Tc_R1, done by using the program MultAlin.

AMPt (F1-R1)

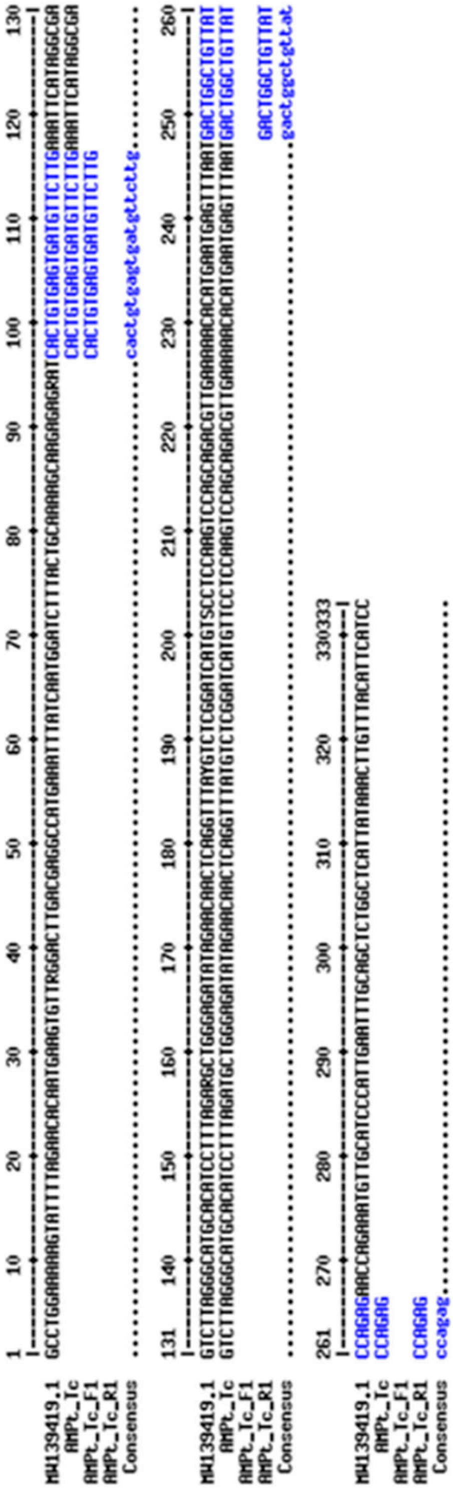

Alignment between the complete sequence of AMPt gene (MW139419.1), the fragment of 170 bases amplified by *T. coccinea*'s cDNA, the forward oligonucleotide AMPt_Tc_F1 and the reverse oligonucleotide AMPt_Tc_R1, done by using the program MultAlin.

ATPs (F1-R1)

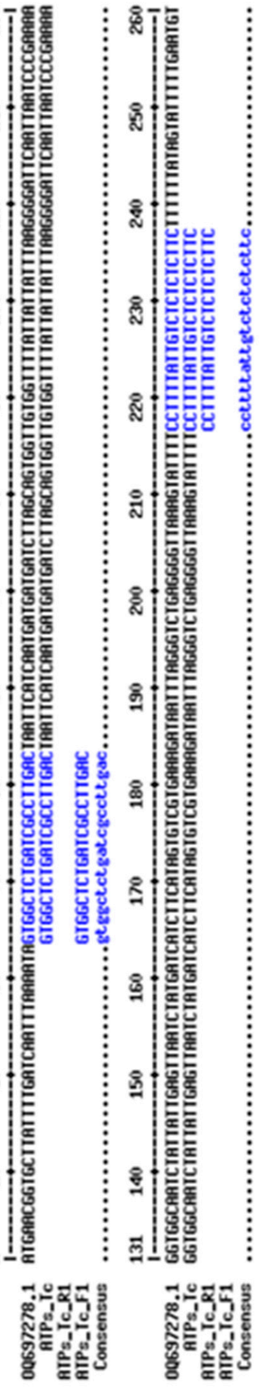

Alignment between the complete sequence of ATPs gene (OQ697278.1), the fragment of 204 bases amplified by *T. coccinea*'s cDNA, the forward oligonucleotide ATPs_Tc_F1 and the reverse oligonucleotide ATPs_Tc_R1, done by using the program MultAlin.

BETA-ACT (F1-R1)

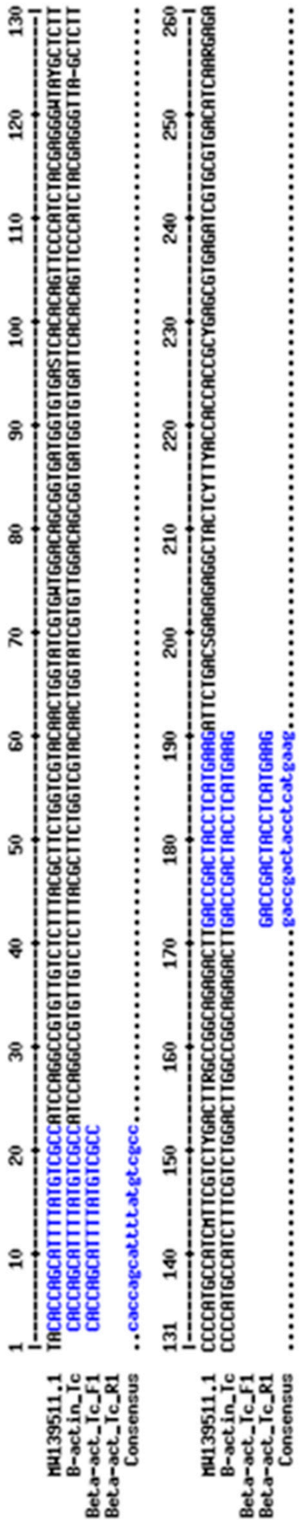

Alignment between the complete sequence of Beta-act gene (MW139511.1), the fragment of 178 bases amplified by *T. coccinea*'s cDNA, the forward oligonucleotide Beta-act_Tc_F1 and the reverse oligonucleotide Beta-act_Tc_R1, done by using the program MultAlin.

Cb (F1-R1)

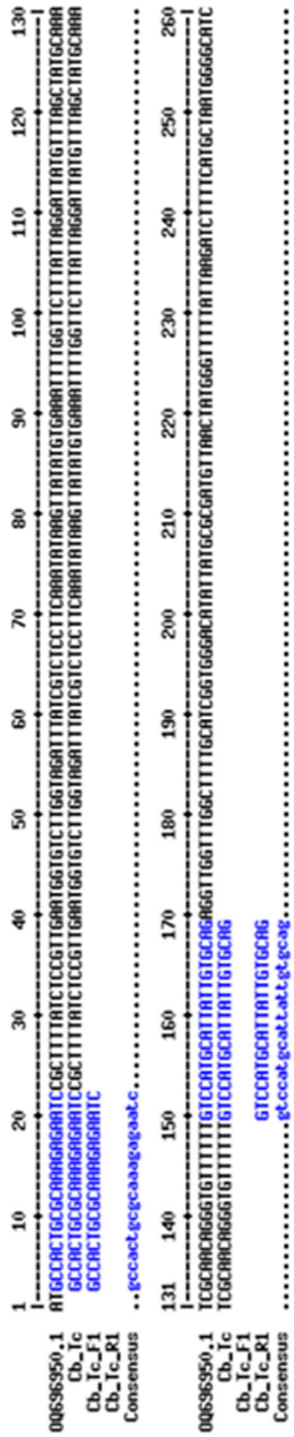

Alignment between the complete sequence of Cb gene (OQ696950.1), the fragment of 167 bases amplified by *T. coccinea*'s cDNA, the forward oligonucleotide Cb_Tc_F1 and the reverse oligonucleotide Cb_Tc_R1, done by using the program MultAlin.

NADH5 (F1-R1)

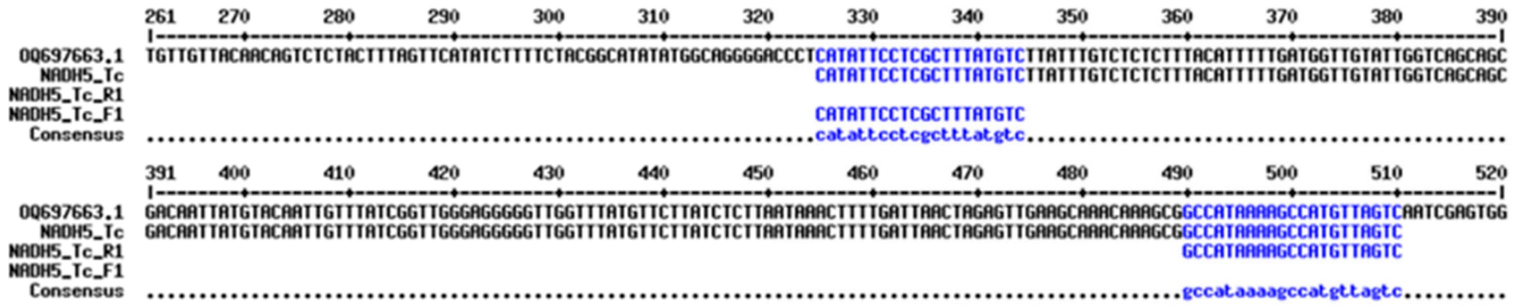

Alignment between the complete sequence of NADH5 gene (OQ697663.1), the fragment of 188 bases amplified by *T. coccinea*'s cDNA, the forward oligonucleotide NADH5_Tc_F1 and the reverse oligonucleotide NADH5_Tc_R1, done by using the program MultAlin.

NADHox (F1-R1)

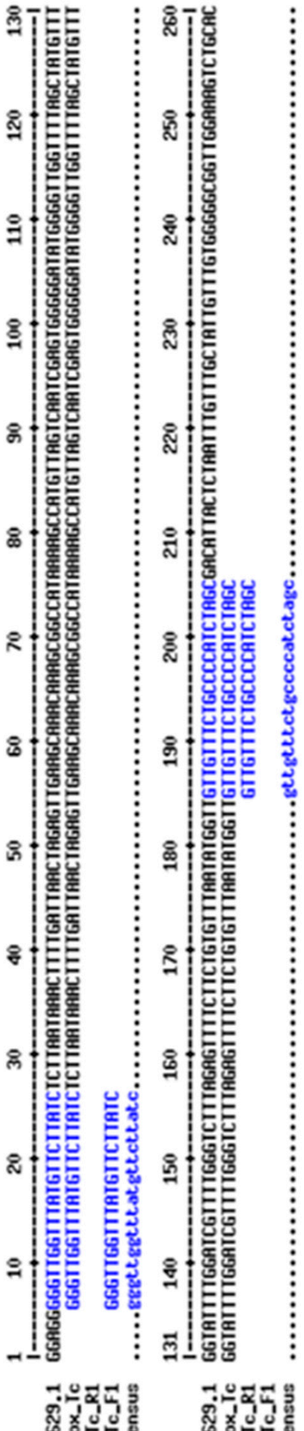

Alignment between the complete sequence of NADHox gene (MW139629.1), the fragment of 200 bases amplified by *T. coccinea*'s cDNA, the forward oligonucleotide NADHox_Tc_F1 and the reverse oligonucleotide NADHox_Tc_R1, done by using the program MultAlin.

NC (F1-R1)

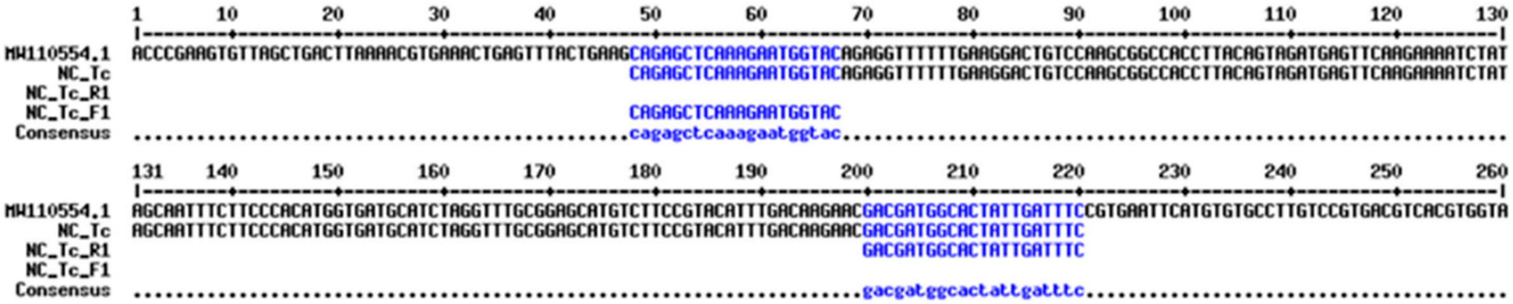

Alignment between the complete sequence of NC gene (MW110554.1), the fragment of 175 bases amplified by *T. coccinea*'s cDNA, the forward oligonucleotide NC_Tc_F1 and the reverse oligonucleotide NC_Tc_R1, done by using the program MultAlin.

Figure S4. Efficiency lines constructed by Real-time qPCR for the genes analyzed of the coral *T. coccinea*.

18S RNA (F1-R1)

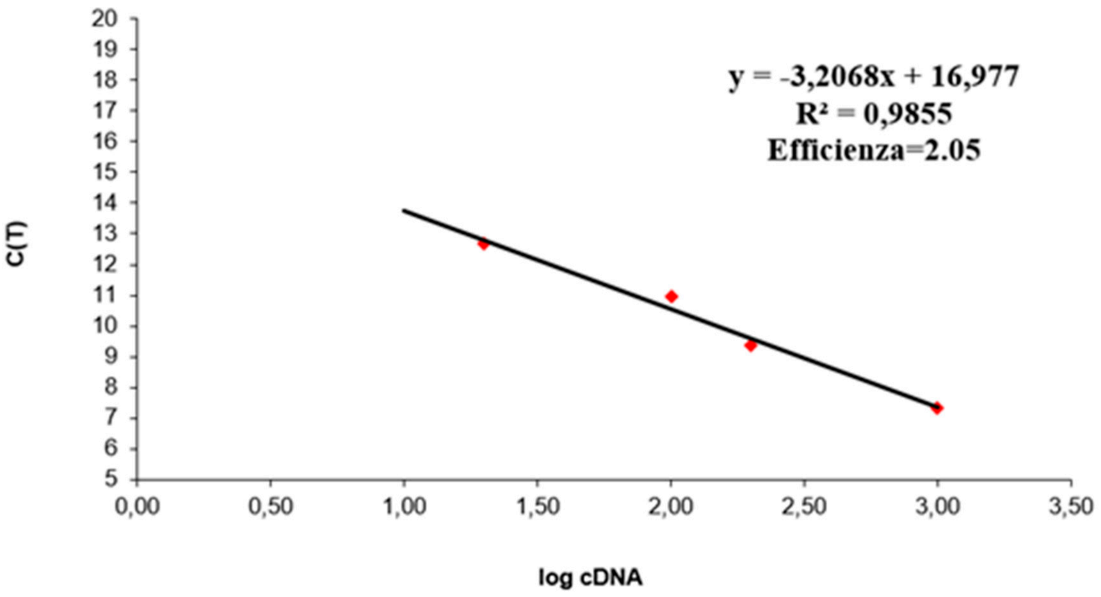

28S RNA (F1-R1)

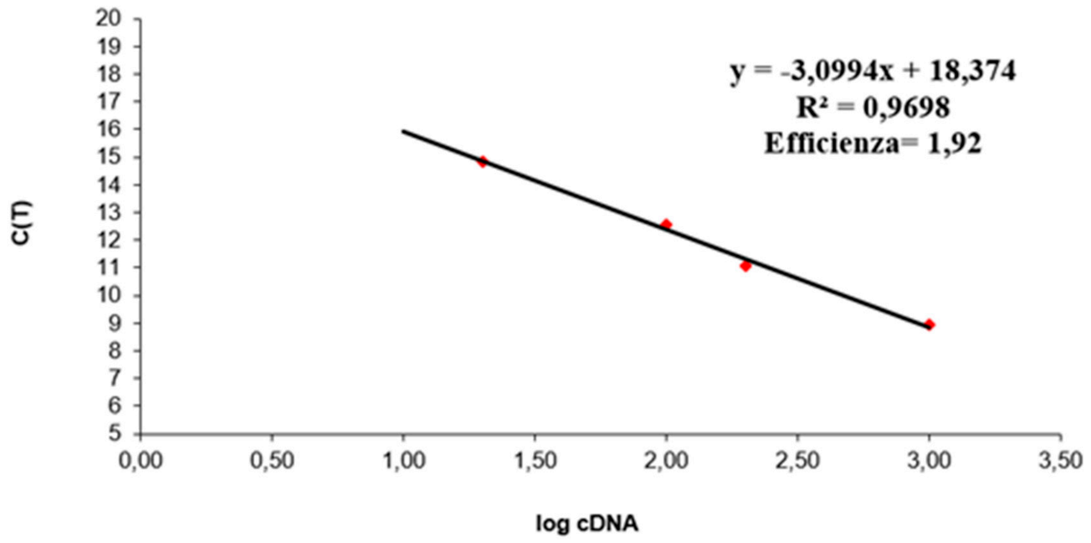

AMPt (F1-R1)

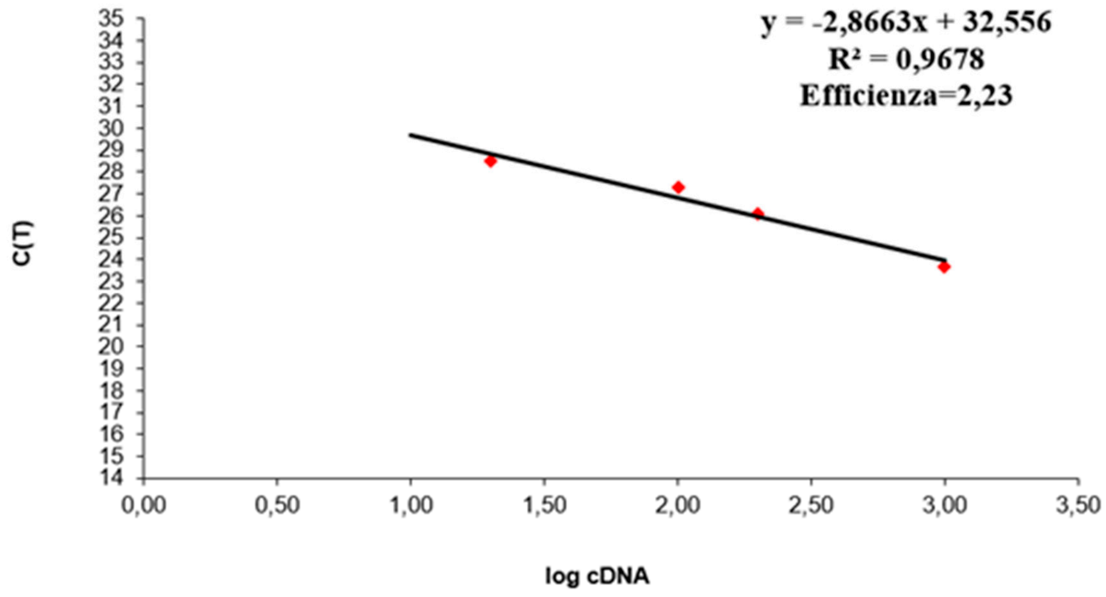

ATPs (F1-R1)

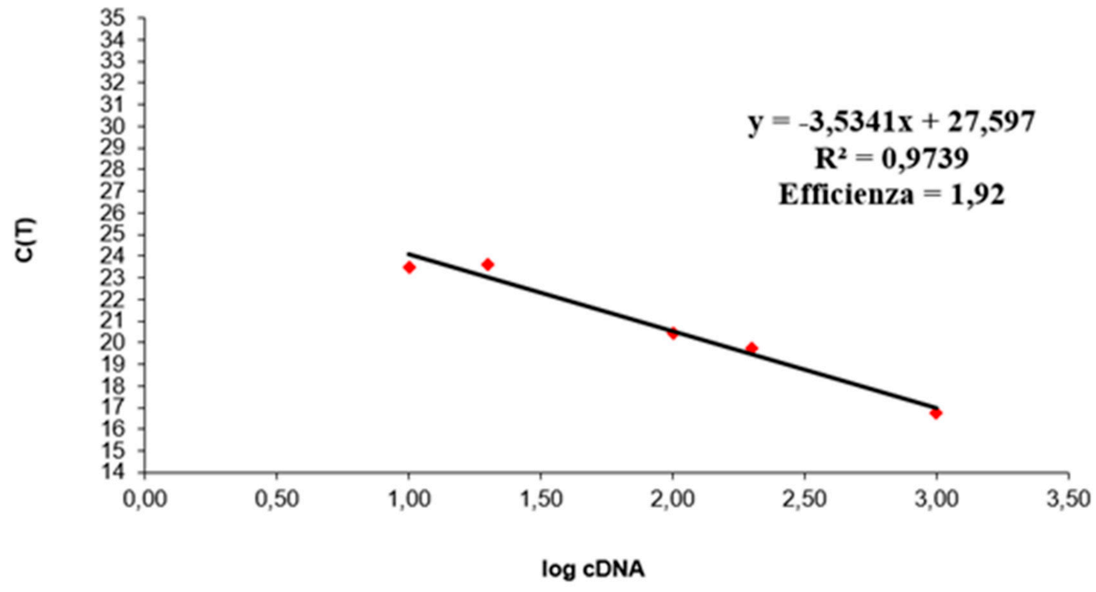

BETA-ACT (F1-R1)

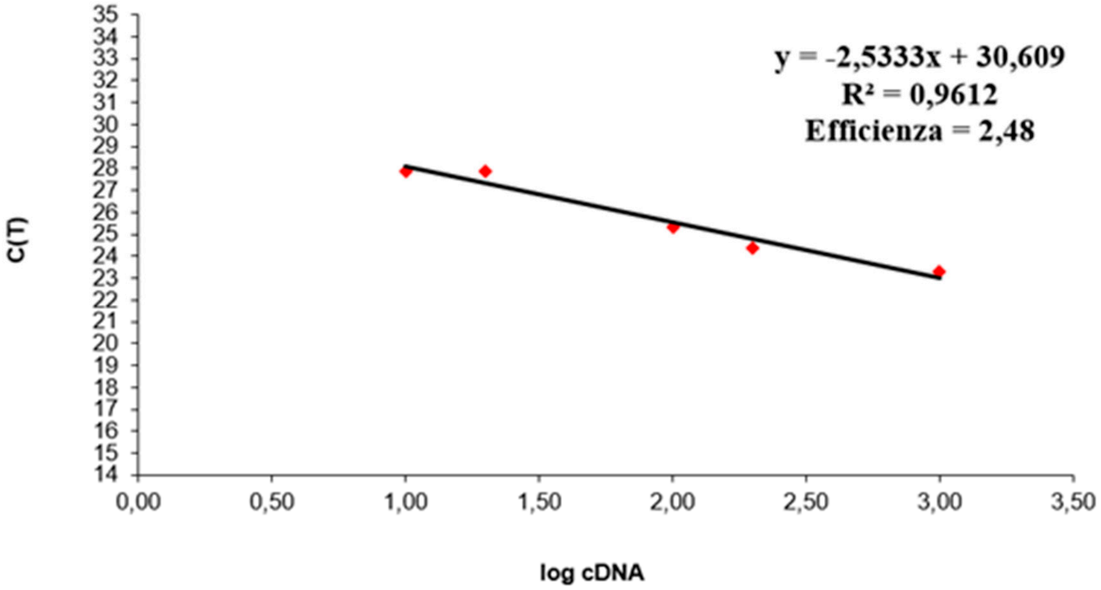

Cb (F1-R1)

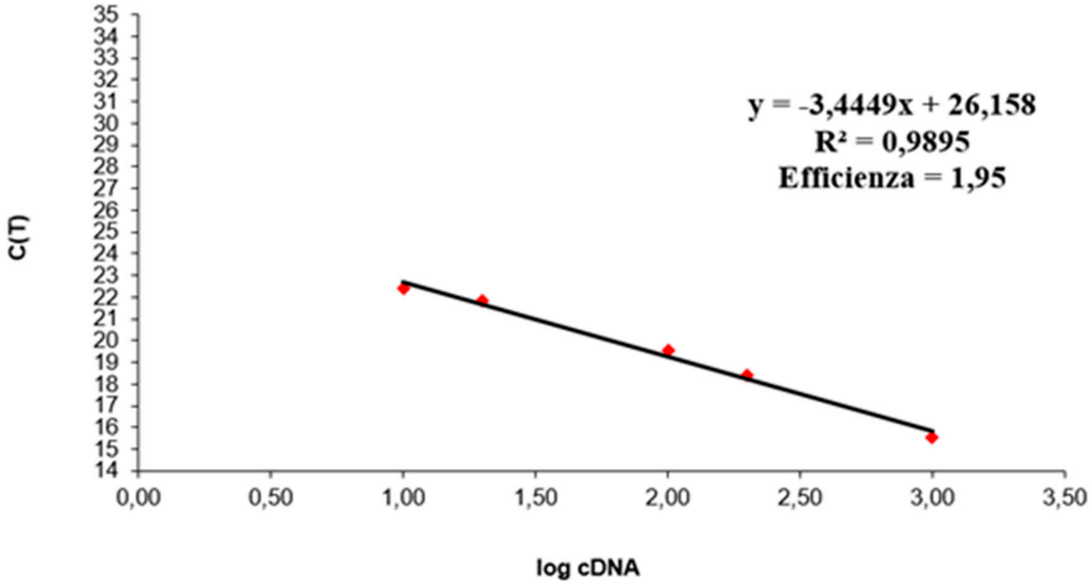

NADH5 (F1-R1)

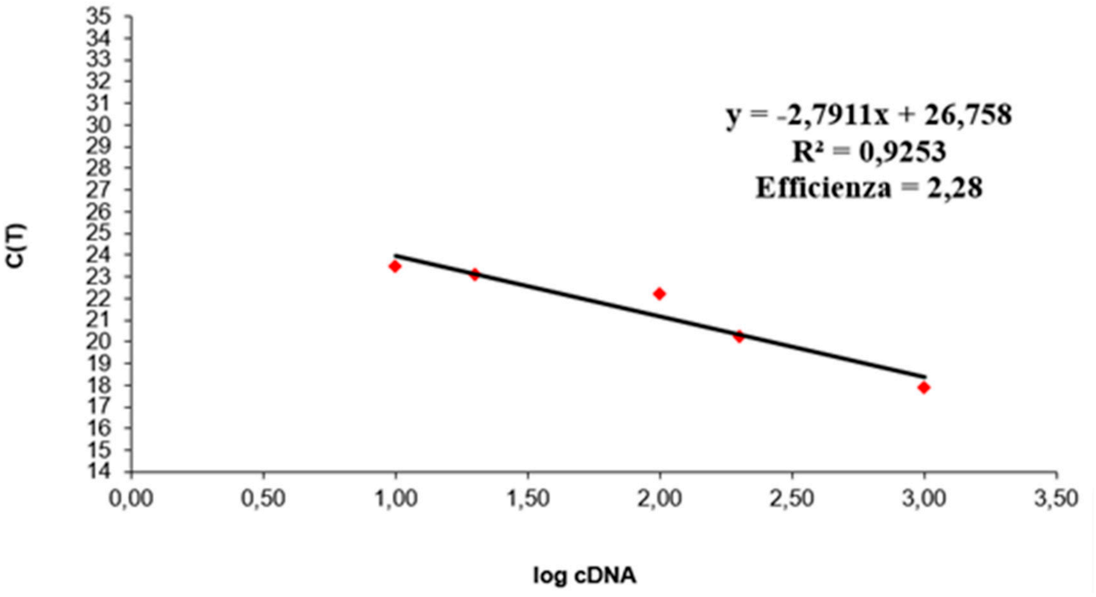

NADHox (F1-R1)

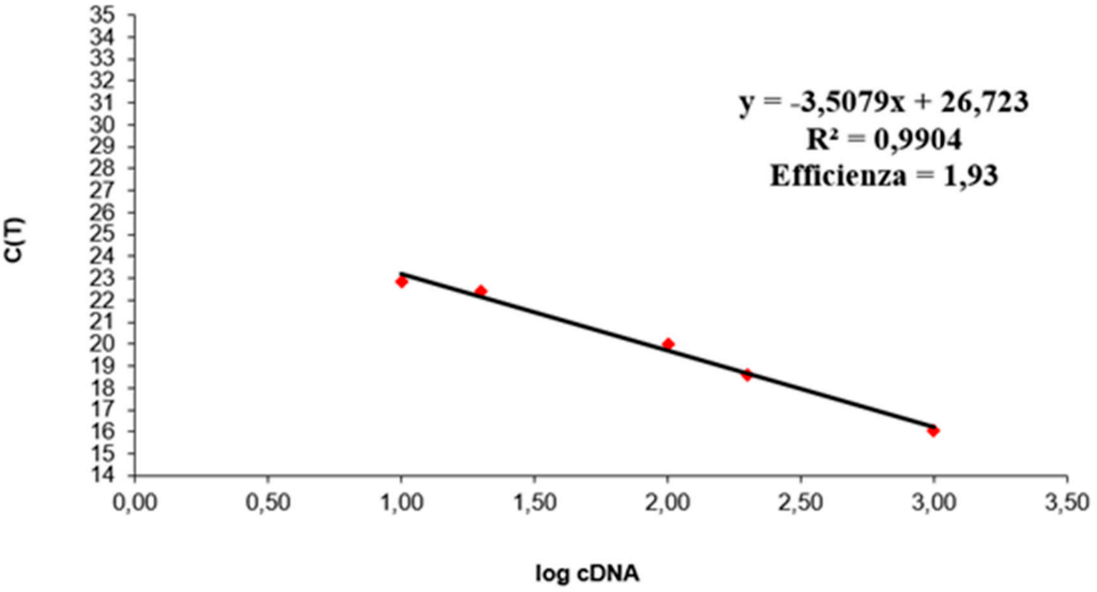

NC (F1-R1)

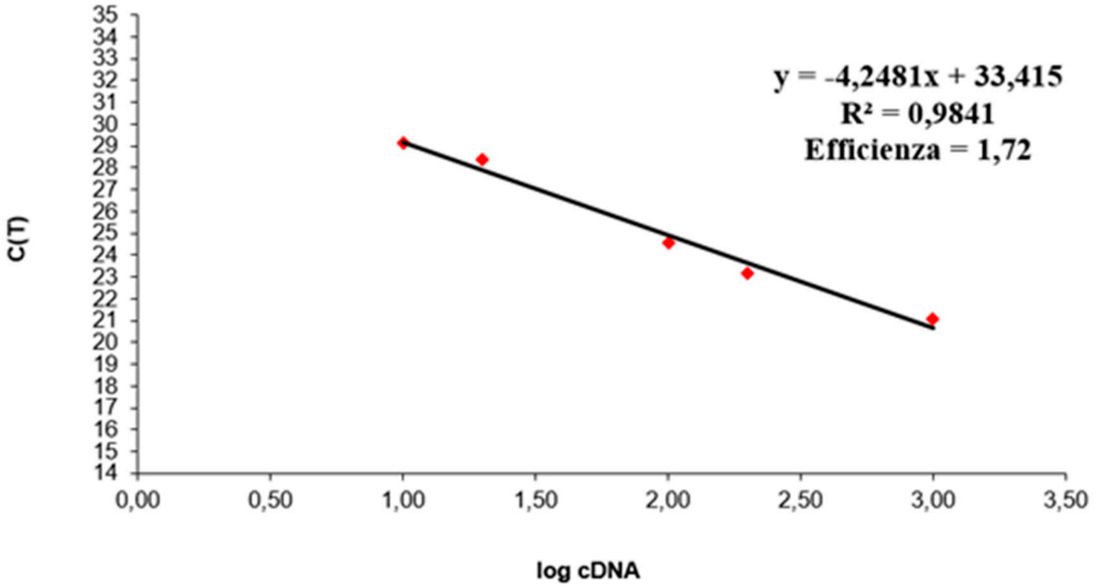

y = -4,2481x + 33,415
R² = 0,9841
Efficienza = 1,72
C(T)
log cDNA
35
34
33
32
31
30
29
28
27
26
25
24
23
22
21
20
19
18
17
16
15
14
0,00
0,50
1,00
1,50
2,00
2,50
3,00
3,50

Table S1. Functional groups and the genes involved in them, as reported by the DAVID analysis. *NCALD* and FICD genes are not currently included in any specific KEGG metabolic pathway.

**KEGG pathway**	**Genes**
Thermogenesis	*ATP5F1A, ACTB, MT-CO1, MT-ND5*
Electron transport coupled proton transport	*ATP5F1A, MT-CO1, MT-ND5*
Oxidative phosphorylation	*ATP5F1A, MT-CO1, MT-ND5, ND1*
Mitochondrion inner membrane	*ATP5F1A, MT-CO1, MT-ND5*

Table S2. Numerical values of gene expression conducted by *Real-Time qPCR* for the seven genes analyzed in polyps from *T. coccinea* and *T. tagusensis* collected from three individuals reared in laboratory conditions respect to the samples directly collected from the field. Fold differences greater than ± 1.5 were considered significant. Values reported in red correspond to up-regulated genes.

**Gene**	***T. coccinea***	***T. tagusensis***
NADH ox	**2,2**	**3,1**
Beta-act	**3,1**	**3,3**
AMPt	**3,2**	**3,6**
NC	**1,6**	**4,2**
NADH 5	**2,1**	**8,9**
ATPs	**2,9**	**2,9**
Cytb	**1,5**	**9,6**
